# Development of environmental DNA metabarcoding primers for marine mollusks and comparison with published primers

**DOI:** 10.1186/s12862-024-02265-8

**Published:** 2024-05-31

**Authors:** Xiaojing Shi, Yihui Jiang, Ling Cao, Cong Zeng

**Affiliations:** 1 School of Oceanography, Shanghai Jiao Tong University, Shanghai, 200030 China; 2 State Key Laboratory of Marine Environmental Science, College of Ocean and Earth Sciences, Xiamen University, Xiamen, 361104 China

**Keywords:** eDNA, Primer development, Mollusk, Metabarcoding, Mitochondrial genes

## Abstract

Monitoring mollusk biodiversity is a great challenge due to their large diversity and broad distribution. Environmental DNA (eDNA) technology is increasingly applied for biodiversity monitoring, but relevant studies on marine mollusks are still limited. Although previous studies have developed several pairs of primers for mollusk eDNA analyses, most of them targeted only a small group of mollusks. In this study, seven primers were designed for the mollusk community and validated and compared with eight pairs of published primers to select the best candidates. After *in silico* test, MollCOI154 and MollCOI255 primers showed non-specific amplification, and same results were also obtained in published primers (COI204, Sepi, and veneroida). Moll12S100, Moll12S195 and Moll16S primers failed to amplify across all genomic DNA from selected mollusk. Except Moll16S, all developed and two published (unionoida and veneroida) primers were successfully amplified on four eDNA samples from Yangtze River estuary. After annotation of the amplified sequences, MollCOI253 showed higher annotation of the amplification results than the other primers. In conclusion, MollCOI253 had better performance in terms of amplification success and specificity, and can provide technical support for eDNA-based research, which will be beneficial for molluscan biodiversity investigation and conservation.

**Supplementary Information:**

The online version contains supplementary material available at 10.1186/s12862-024-02265-8.

## Introduction

Mollusks are the second largest group of invertebrates and are widely distributed in freshwater and marine ecosystems, playing important roles in the ecosystem, such as modifying the sediment and purifying water [1]. Currently, nearly 100,000 species of mollusks have been reported in the world, and they are considered as the key taxa for marine biodiversity in different marine areas [2]. Additionally, mollusks are important components of global fisheries products, and the world production of major molluscan species has shown a steady increase since 1950 [3].On the other side, due to their poor mobility and sensitivity to environmental changes, mollusks are more susceptible to the impacts of global warming and human disturbances compared to other marine organisms [4], which makes it urgent to monitor and protect their biodiversity.

Traditional biodiversity monitoring is mainly based on obtaining data on community composition through trawling, diving and the use of underwater cameras, which are costly, time-consuming and highly dependent on the involvement of taxonomic experts, who are currently becoming increasingly scarce [5]. Therefore, these limitations of traditional survey pose significant challenges to biodiversity research [6]. In recent years, environmental DNA (eDNA) technology has emerged as a novel approach for studying community compositions and species detection in ecological and biodiversity research [7]. Unlike traditional methods, eDNA technology is non-invasive, which does not damage the target species and the ecosystems [8]. Besides, its high specificity and sensitivity make eDNA particularly effective for studying hard-to-capture, invasive, and rare species [9]. With an increasing concern on the marine conservation and sustainable fisheries, eDNA technology is particularly important for the study of aquatic organisms [10]. As the cost of high-through sequencing decreased, the metabarcoding, a combination of DNA barcoding and high-through sequencing, provided solutions for the simultaneous detection of multiple species as well as relative abundance, which is more suitable for community analysis. Environmental DNA-based metabarcoding (eDNA metabarcoding) technology was expected to further improve the detection efficiency of aquatic organisms and contribute to the establishment of a standardized monitoring technology system for freshwater and marine ecosystems.

While in recent years eDNA technology has been applied to mollusk studies, existing primers target specific groups, such as Unionida and Venerida in freshwater and Cephalopoda in marine ecosystems [10]. A total of eight primer pairs were developed in previous publications for molluscan eDNA detection or diversity analysis, of which four primers (NZMS, NADH, COI204, and Sepi) were used for single-species detection, and four primers (16 S rRNA, unionoida, veneroida, and Ceph18S) were used for diversity analysis. Among the single-species target primers, *Cytochrome b* (*Cytb*) [11], *NADH* [12] and *Cytochrome c oxidase* subunit 1 (*COI*) [13, 14] gene were the candidate regions for primer design. For diversity analysis, metabarcoding primers (16 S rRNA, unionoida, veneroida and Ceph18S) were reported to be able to be used in a wide range of molluscan community analyses [15–17], and these primers were mainly targeting *16 S* and *18 S* rRNA regions. However, 16 S rRNA, unionoida and veneroida primers were applied in the analyses of freshwater bivalves but not in marine mollusks, and Ceph18S was only designed for the biodiversity study of cephalopod species [18]. Since most mollusks inhabit the marine environment, further development of eDNA primers for mollusks is still required to include more species to meet the urgent needs of mollusk biodiversity surveys.

The aim of this study was to develop new environmental DNA primers and evaluate their effectiveness with those already developed in previous studies. For this, Chinese offshore mollusks were selected as candidates for primer design and testing, and the mitochondrial genes *COI*, *12s* and *16 S* were selected as target gene regions. The amplification performance of designed primers from different gene regions, as well as published primers, were compared by *in silico* PCR, genomic DNA amplification and eDNA amplification. The results are expected to identify suitable primers for environmental DNA-based mollusk biodiversity surveys, contributing to future marine biodiversity conservation efforts.

## Materials and methods

### Sequence collection for primer design

Given the vast diversity and uneven distribution of mollusks, the specificity (number of non-target species amplified), universality (number of target species amplified) and robustness (amplification success rate) of the developed primers were mainly tested by mollusks from the coastal areas of China. Candidate species were selected based on the *Atlas of Marine Mollusks in China* [3], and further checked the existence of genetic sequences of these species in NCBI database (https://www.ncbi.nlm.nih.gov/). To ensure sufficient sequence length for primer region screening, only species with complete mitochondrial genomes in the NCBI database were chosen. After screening, a total of 213 molluscan species were obtained and their sequences were downloaded from NCBI using Geneious (versionR11) (https://www.geneious.com/). Based on previous studies and initial alignments, the full-length gene regions of *COI*, *12 S* and *16 S* were selected as candidate regions for primer design. Multiple alignments for these three genes across the 213 species were performed using MAFFT alignment in Geneious. Alignments were also further visualized using Geneious [18], and the visualized alignments were then used to identify primer binding regions and variant sites.

### Primer development

Primer regions were identified considering three crucial requirements: (1) because most eDNA is easily degraded and the sequencing platform (mainly Illumina) usually requires the sequencing fragments to be shorter than 500 bp [19], and further shortened due to the additional adapters at both ends of the amplified fragments required during sequencing and library construction. As such and based on previous studies, in which the amplification products of environmental DNA usually did not exceed 300 bp in length and to obtain a sufficient number of variable sites within the sequence variants, the length of the target region was set to be between 100 and 300 bp [19]; (2) to provide a good taxonomical resolution, the target regions should include sufficient inter-specific DNA variation for all target species [20]; and (3) to successful conduct PCR amplification, conservative regions for binding PCR primers (more than 18 bp in length) across all target species should be located at both ends of the targeted regions [21]. Primers were designed using Geneious accounting for G/C contents (40–60%), melting temperature (Tm: 50–60 °C), primer length (18–27 bp) and product size (100–300 bp).

### Analysis of *in silico* PCR

The mollusk metabarcoding developed primers were evaluated using *in silico* PCR implemented in the Primer-BLAST program from NCBI website (https://www.ncbi.nlm.nih.gov/tools/primer-blast/index.cgi?LINK_LOC=BlastHome). Specific settings included: 1) PCR product size within 100–500 bp; 2) primer melting temperatures between 57℃-63℃; 3) a maximum Tm difference of 2℃ between forward and reverse primers; 4) a maximum of 3 mismatches between each primer and the target sequence; and no mismatches in the last two nucleotides at the 3’ end of the primer [22]. The Primer-BLAST search was conducted against the nr nucleotide database in NCBI. Specificity, universality and robustness results of *in silico* PCR were compared among developed and published primers.

### Tests in genomic DNA amplification

To further confirm the universality of developed primers, genomic DNA was extracted from 24 species across three mollusk classes: 6 Gastropoda (*Haliotis discus*, *Nodilittorina pyramidalis*, *Babylonia lutosa*, *Neptunea cumingi*, *Rapana bezoar*, *Rapana rapiformis*), 16 Bivalvia (*Mactra quadrangularis*, *Mactra veneriformis*, *Mactra antiquata*, *Mactra chinenesis*, *Tegillarca granosa*, *Scapharca subcrenata*, *Paphia undulata*, *Meretrix meretrix*, *Grassostrea gigas*, *Azumapecten farreri*, *Mytilus edulis*, *Perna viridis*, *Musculus senhousei*, *Trichomya hissutus*, *Sinonovacula constricta*, *Solen strictus*) and 2 Cephalopoda (*Octopus vulgaris*, *Loligo chinensis*). In addition, two decapod species, *Penaeus monodon* and *Ocypoda ceratophthalma*, from the East China Sea, were also selected to verify the specificity of the mollusk primers through PCR amplification. Also, according to previous studies, 16SrRNA primer had a better performance [15] and it was also selected to test in all specimens. Total genomic DNA was extracted from each species, preserved in 95% ethanol, using the MolPure Cell/Tissue DNA Kit (Yeasen Biotechnology, China). The extracted DNA was stored in -20 ℃.

PCR was carried out in a 50.0 µl reaction volume containing 20.0 µl sterile distilled water, 25.0 µl 2 × Gflex PCR buffer, 2.0 µl of each primer, and 1.0 µl DNA template. The thermal cycle profile after an initial 5 min denaturation at 94℃ was as follows: denaturation at 94℃ for 30 s, annealing at 53℃ for 30 s and extension at 72℃ for 30s with the final extension at 72℃ for 10 min. The PCR products were checked by 1.80% agarose gel electrophoresis to check whether the amplification was successful. The amplified products were further sequenced for verification by Tsingke Biotechnology Co. Ltd. (Beijing, China).

### Environmental DNA amplification tests

To test *in-situ* sample amplification, four seawater samples were collected at Yangtze Estuary in the East China Sea, where the presence of mollusks is well documented. Sampling was conducted at four sites (30.70°N 123.21°E, 31.32°N 121.75°E, 29.17°N 122.77°E, 28.75°N 122.47°E) covering Yangtze estuary onboard of R/V “Zheyuke2” and “Runjiang1” implementing the open research cruise NORC2021-03. Water sampling was collected from October to November 2021.

For environmental DNA collection, 1 L water were collected at each site from the bottom layer (1–3 m above the seabed) and stored at 4 ℃. Water samples were first filtered through a 1 μm glass microfiber filter (Whatman, UK) to remove the large particles and organisms, and then filtered through a 0.45 μm mixed-fiber filter (Jinteng, China) within 10 h after sampling. The filter papers were preserved in ethanol at -20 ℃. The eDNA extraction, library construction and pair-end 2 × 250 bp sequencing were completed in Shanghai Personal Biotechnology Co., Ltd. (Shanghai, China).

Bioinformatics analyses were mainly performed with QIIME 2 (2023.2) [23]. Briefly, raw sequence data were demultiplexed using the demux plugin followed by primers cutting with cutadapt plugin [24]. Sequences were then merged, filtered and dereplicated using functions of fastq_mergepairs, fastq_filter, and derep_fulllength in Vsearch. All the unique sequences were then clustered at 98% (via cluster_size) followed by chimera removing (via uchime_denovo). Finally, the non-chimera sequences were re-clustered at 97% to generate Molecular Operational Taxonomic Unit (MOTU) representative sequences and MOTU frequency table, and the MOTU clustering procedure following the Vsearch (v2.13.4) [25]. Taxonomy was assigned to MOTUs using BROCC [26] against the mollusk sequences from mollusk nr Database in NCBI (accessed in March 2023).

## Results

Based on the selection criteria, a total of 213 mollusk species were kept for primer design, including 125 bivalves, 27 cephalopods, and 61 gastropods. Three gene sequences, *COI*, *12 S* and *16 S*, were selected from the mitochondrial genomes of these mollusks, and the sequences of these three genes were separately aligned to identify conservative regions. After alignment, seven pairs of primers were designed from these regions, including three pairs from *COI*, three pairs from *12 S* and one pair from *16 S*. To find the most effective primers, these seven primer pairs, as well as eight previously published primer, were analyzed to compare their amplification performance.

New developed primers.

**Fig. 1:**
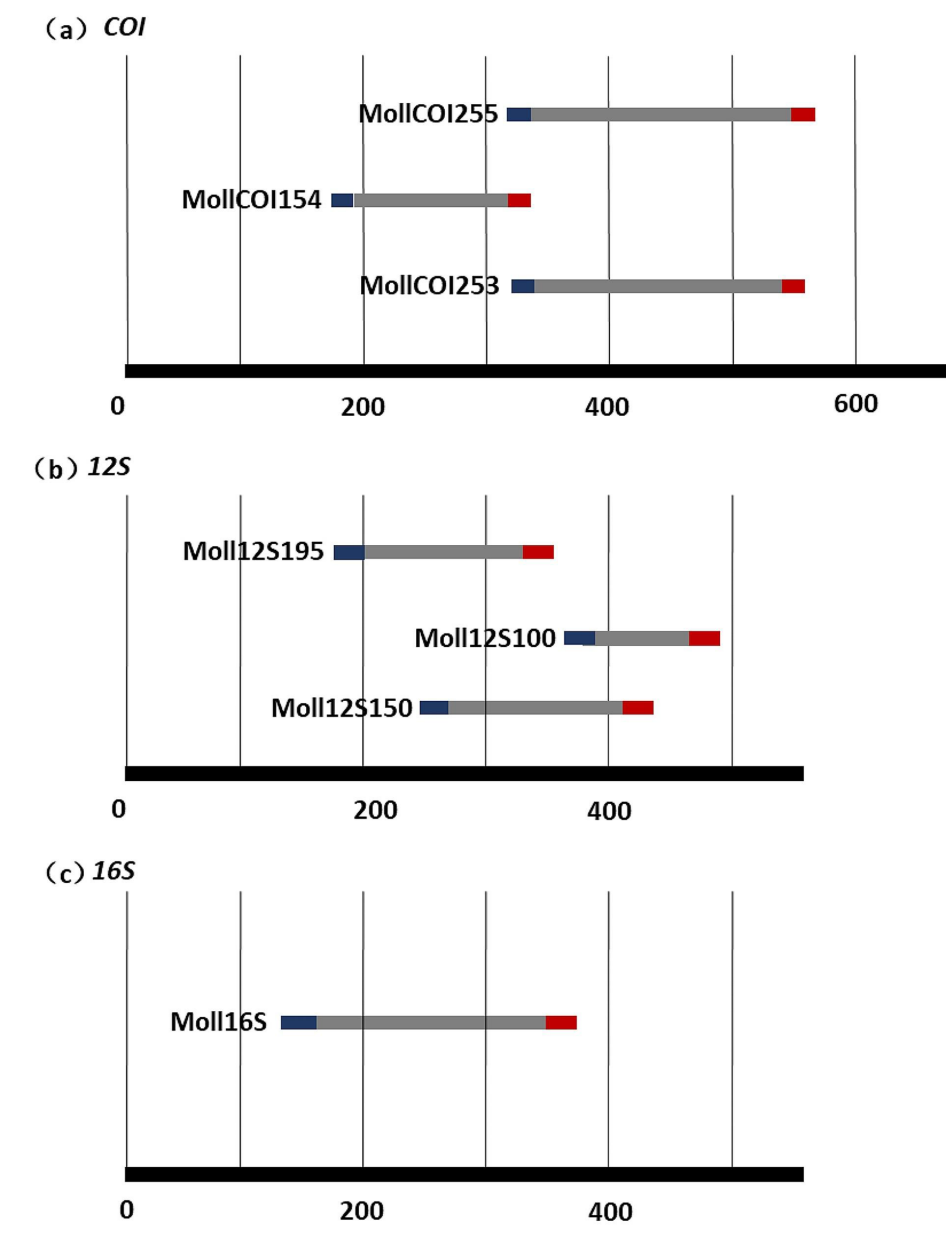
Target amplification regions of new primers for *COI***(a)**, *12 S***(b)** and *16 S***(c)**. The black lines are the three genes and the numbers below the lines are the sequence lengths. The gray line is the target amplification region, and the blue and red fragments at the ends of the gray line are forward and reverse primers

Seven primer pairs were identified, each targeting a conservative region flanking a hyper-variable region within the respective genes. In the *COI* region, three primers, MollCOI253, MollCOI154 and MollCOI255, were developed, and all these primers shared a conservative region (Fig. 1a; Table 1). All three pairs exhibited an annealing temperature of approximately 57˚C, with PCR product lengths ranging from 154 to 255 bp. For the *12 S* region, Moll12S150, Moll12S195 and Moll12S100 were designed, and all these primers did not share binding regions (Fig. 1b; Table 1). The annealing temperature and PCR product lengths for these primers varied from 56 to 59 ˚C and 100–195 bp, respectively. The primer Moll16S was the only primer designed targeting the *16 S* gene, and the annealing temperature and PCR product lengths were 60 ˚C and 228 bp, respectively (Fig. 1c; Table 1). These seven primer pairs basically met the eDNA amplification requirements in terms of length and annealing temperature.

**Table 1:** Primers tested in this study

Target gene	Primer name	Primers (5’~3’)	Product size(bp)	Annealing Temperature	GC%	Source
*COI*	MollCOI*154*	F-TGGGGGTTTTGGTAATTGGT	154	57˚C	45.00	Present study
		R-TCAACCAGTACCAGCTCCA			52.63	
	MollCOI253	F-GGAGTAGGAACTGGTTGGAC	253	57˚C	55.00	
		R-CAGCTGCTAACACAGGCA			55.56	
	MollCOI255	F-TGGAGCTGGTACTGGTTGA	255	57˚C	52.63	
		R-GCCCCAGCTAAAACAGGTAT			50.00	
*12 S*	Moll12S100	F-TTGTATACCGTCGTCGTCAG	100	57˚C	50.00	
		R-AGCTGCACCTTGATCTGAC			52.63	
	Moll12S150	F-TATGCTTGCCGGGCAACT	150	59˚C	55.56	
		R-ACGGCCATACACCAACTGA			52.63	
	Moll12S195	F-GTATTGCCGTTGTCAGCTT	195	56˚C	47.37	
		R-CCTTACTCCTAAGTTCACCTTC			45.45	
*16 S*	Moll16S	F-GTCCTGTGAATGGTTTGACGAG	228	60˚C	50.00	
		R-TTGCTGCCCCAGCCAAAAC			57.89	
*COI*	COI204	F-TGTTACAGCTCACGCATTTGTT R-CCGGTACCTGCACCTCTTTC	204	60˚C	40.91	[13]
					60.00	
	Sepi	F-CACCAGACATAGCCTTCC	155	54˚C	55.56	[14]
		R-GCCAGCATGAGATAGATTAC			45.00	
*NADH*	NADH	F-TCGAGCCATAGCTCAAACCA	147	59˚C	50.00	[12]
		R-GCGAGTGGTAGTGAAAGAGT			50.00	
*Cytb*	NZMS	F-TGTTTCAAGTGTGCTGGTTT	92	56˚C	40.00	[11]
		R-CAAATGGGCTAGTTGATTCTTT			36.36	
*16 S*	16 S rRNA	F-TGAGCGTGCTAAGGTAGC	360	60˚C	55.56	[15]
		R-AGCCAACATCGAGGTCGC			61.11	
	unionoida	F-GCTGTTATCCCCGGGGTAR	170	58˚C	61.11	[16]
		R-AAGACGAAAAGACCCCGC			55.56	
	veneroida	F-CSCTGTTATCCCYRCGGTA	159	56˚C	52.63	[16]
		R-TTDTAAAAGCCGAGAAGACCC			42.86	
*18 S*	Ceph18S	F-CGCGGCGCTACATATTAGAC	173–235	61˚C	55.00	[17]
		R-GCACTTAACCGACCGTCGAC			60.00	

### Tests of *in silico* PCR amplification

**Fig. 2:**
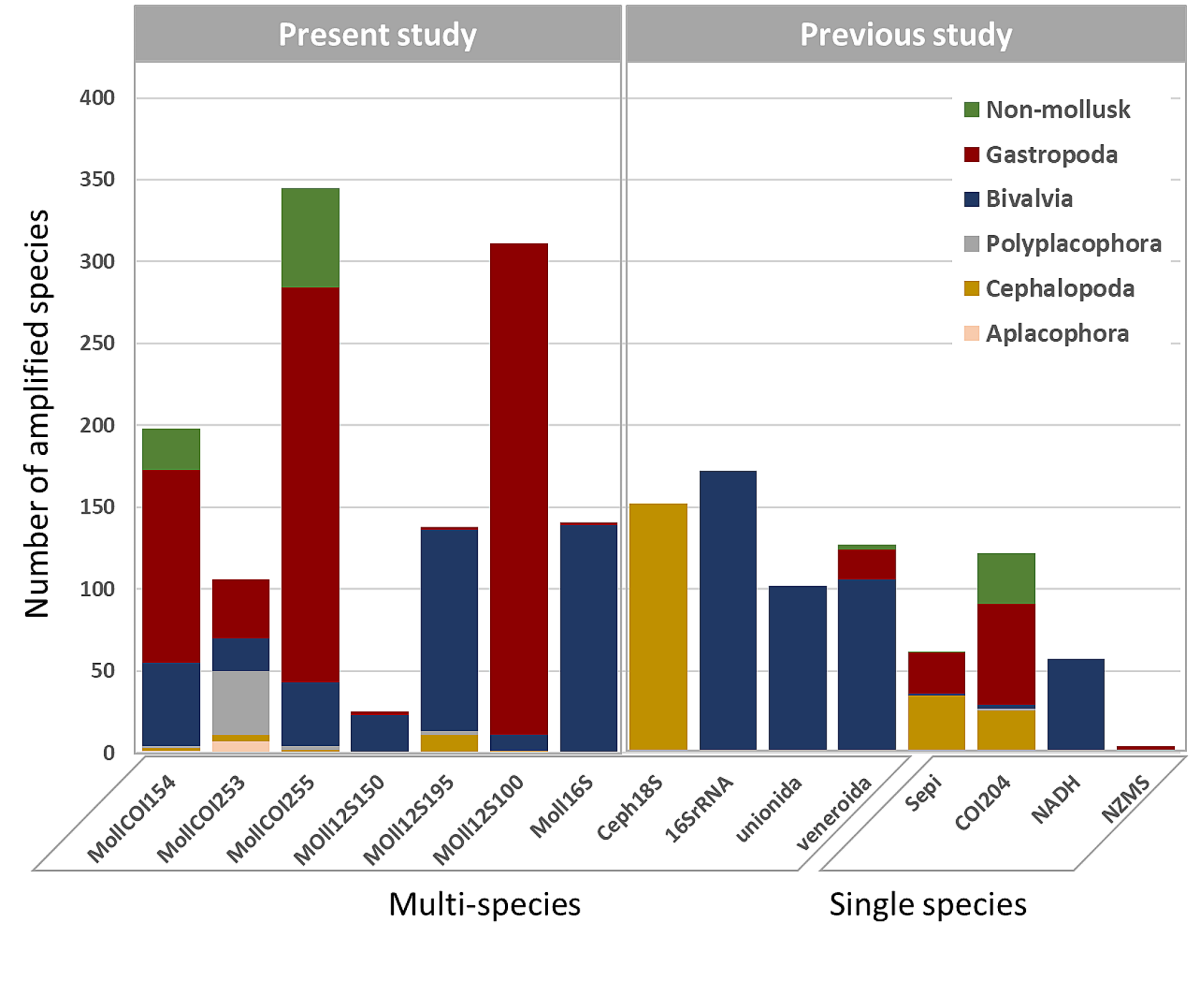
Comparison of new primers and previous primers *in silico* PCR amplification results

Numbers of amplified species from *in silico* PCR amplification were presented in Table S1 for both newly developed and published primers, and the results showed that robustness, specificity and universality varied considerably among the primers assessed. Among the newly developed primer pairs, MollCOI255 had the highest robustness, while Moll12S150 had the lowest (Fig. 2). Both MollCOI154 and MollCOI255 amplified non-molluscan taxa with a not insignificant percentage of 12.63% and 17.68%, respectively, indicating that these two pairs of primers had less specificity (Table S1). Furthermore, distinct amplification preferences were observed for different primers, except for MollCOI253. The MollCOI255 primers and Moll12S100 amplified more gastropods (69.86% and 96.46%, respectively) (Table S1), while the other primers favored bivalves. Among these primers, only MollCOI154 and MollCOI253 were successful in amplifying all classes of mollusks (Table S1).

Among reported primers, NADH (targeted *Lampsilis siliquoidea*), 16SRNA (targeted Bivalvia) and unionida (targeted Unionida) exclusively amplified bivalves, while Ceph18s (targeted Cephalopoda) exclusively amplified cephalopods, suggesting that these primers had a high specificity but low universality for mollusks (Table S1). COI204 (targeted *Octopus vulgaris*), Sepi (targeted Cephalopoda) and veneroida (targeted Veneroida) primers demonstrated broader mollusk coverage but they also amplified non-molluscan taxa, particularly COI204 (25.41% non-mollusks). NZMS, although it exhibited good specificity, had the lowest species coverage among all examined primers (Fig. 2). Due to their lack of specificity in *in silico* PCR test, COI204, MollCOI154 and MollCOI255 were excluded from genomic and environmental DNA amplification assays.

### Testing developed primers using genomic DNA

After PCR amplification with genomic DNA, 16SrRNA was successfully amplified in all tested species, indicating the capability and suitability of DNA templates for assessing primer amplification (Table 2; Table S2). Apart from 16SrRNA, Moll12S150 and MollCOI253 primers successfully amplified all 24 mollusks from 15 families, and Moll12S100 was the least successful (45.83% success rate). Moll12S195 amplified 66.67% and 75.00% of the genomic DNA successfully for Gastropoda and Bivalvia, respectively, whereas Moll16S amplified these two Classes less successfully (50.00% and 43.75%). In addition, neither primer pair was able to amplify cephalopods. In contrast, Moll12S100 was able to amplify the all sample of gastropods and cephalopods, while bivalves were amplified very inefficiently (18.75%). These differences reflected amplification bias between primers. None of the examined primers amplified decapods except 16SrRNA, which further implied that these primers had a high specificity (Table 2). Sanger sequencing confirmed that the PCR product lengths of all tested primers closely matched the expected lengths, and further BLAST of the sequenced PCR products against the nr database did not reveal any non-target regions of amplification.

**Table 2:** Primer validation results based on genomic DNA amplification

Primer name	Bivalvia	Cephalopoda	Gastropoda	Non-mollusk
MollCOI253	16/16 (100%)	2/2 (100%)	6/6 (100%)	0
MOll12S150	16/16 (100%)	2/2 (100%)	6/6 (100%)	0
MOll12S195	12/16 (75%)	0	4/6 (75%)	0
MOll12S100	3/16 (18.75%)	2/2 (100%)	6/6 (100%)	0
Moll16S	7/16 (43.75%)	0	3/6 (50%)	0
16SrRNA	16/16 (100%)	2/2 (100%)	6/6 (100%)	2/2 (100%)

### Testing developed primers using environmental DNA

After extraction of the eDNA, five designed primers (MollCOI253, Moll12S150, Moll12S100, Moll12S195, Moll16S) were tested using eDNA PCR amplification. To better compare the effect of the newly developed primers, two pairs of published metabarcoding primers (unionoida and veneroida) were also used for amplification and high-throughput sequencing. Moll16S failed to amplify and perform the sequencing library construction, while the rest of the primers successfully passed quality control for high-throughput sequencing. In addition, the results of two samples of the high-throughput sequencing for Moll12S195, Moll12S100, unionoida and veneroida had very few reads, so the results for these two samples were not included in the analysis as well.

The high-throughput sequencing data for MollCOI253, Moll12S150, Moll12S195 and Moll12S100 amplicons comprised a total of 116,750, 351,475, 567,992 and 384,919 raw reads, respectively, which generated 485, 8031, 952 and 6 MOTUs. After annotation using the nr database, 13,749 reads of MollCOI253 amplicon were mollusk (11.78%), and 12 species were detected, of which the largest proportion was *Sulcospira paludiformis* (3.93% of total abundance, including MOTUs that could not be annotated), followed by *Mopalia spectabilis* (3.54%) and *Choanomphalus hyaliniiformis* (2.42%) (Table 3, Table S2). For the Moll12S150 amplicon, 418 reads were annotated as mollusks (0.12%), and 3 species were found, including *Crassostrea ariakensis* (0.11%), *Radix acuminata* (0.003%) and *Doryteuthis opalescens* (0.002%) (Table S3). However, Moll12S195 amplicon had only 9 reads identified as mollusks, belonging to Lucinidae. Moll12S100 amplicon did not yield any annotated mollusk species across all MOTUS in the four samples (Table 3). Although most MOTUs from developed primers could not be annotated to specific mollusk species, those that were annotated largely represent mollusk species that have been previously reported from coastal areas of China.

**Table 3:** High-throughput sequencing results based on environmental DNA amplification

Primer name	Samples	Raw reads Total (range)	MOTUs	Mollusks %	No. Annotated Mollusks	Source
MollCOI253	4	116,750 (23,747 − 35,831)	4,85	11.78%	12	Present study
Moll12S150	4	351,475 (83,344 − 92,908)	8,031	0.12%	3	
Moll12S195	2	567,992 (198,732 − 369,260)	952	0.001%	1	
Moll12S100	2	384,919 (159,907 − 225,012)	6	0	0	
unionoida	2	139,868 (68,264 − 71,604)	347	0	0	
veneroida	2	225,221 (109,582 − 115,639)	1,521	0	0	
16SrRNA	6	461,185	-	26.69%	8	[15]
Ceph18S	19	9,936,758 (23,311 − 50,066)	-	-	15	[40]
Unionoida + veneroida	15	-	-	-	42	[16]

Note: - meant information was not available in the publications

When comparing the annotation effect of all the primer amplicons, it was found that none of the amplification products were found to contain Aplacophora, which could mean that none of these primers were able to amplify Aplacophora or that the sampling point region did not have Aplacophora. In addition, the results of amplification showed that most of examined primers also amplified Cephalopoda and Polyplacophora poorly, with only one pair of primer products detecting them (Table 4). Unlike the developed primers, even though amplifications of unionoida and veneroida was successful and relatively good sequencing results were obtained, the majority of MOTUs were annotated as microorganisms (mainly bacteria). With the exception of Moll12S100, all developed and published primers had a relatively large proportion of products that could not be annotated (42–93%), implying that the use of the nr database as a reference data for mollusks remained insufficient (Table 4).

Based on the results of eDNA amplification and annotation, MollCOI253 had a relatively high amplification success rate (all samples succeeded), and produced raw reads and MOTUs similar to those reported in other studies. Although the percentage of non-annotatable MOTUs was still not low, similar to the other primers, it could be improved by constructing specific annotation database. Since none of the examined primers provided a perfect solution, it can be assumed that MollCOI253 is a relatively good candidate primer for molluscan eDNA studies.

**Table 4:** Amplicon annotation results for the examined primers after pooling all samples

Taxon	MollCOI253	Moll12S150	Moll12S195	Moll12S100	unionoida	veneroida
Aplacophora	0	0	0	0	0	0
Cephalopoda	0	1 (7, 0.00%)	0	0	0	0
Polyplacophora	1 (4,138, 3.54%)	0	0	0	0	0
Bivalvia	2 (611, 0.52%)	14 (400, 0.11%)	1 (9, 0.00%)	0	0	0
Gastropoda	15 (9,000, 7.71%)	1 (11, 0.00%)	0	0	0	0
Non-mollusk	70 (3,746, 3.21%)	1,036 (82,214, 23.39%)	101 (40,203, 7.08%)	6 (384,919, 100%)	324 (14,057, 10.05%)	406 (131,022, 58.17%)
Unassigned	397 (99,255, 85.02%)	6,979 (268,843, 76.49%)	850 (527,780, 92.92%)	0	23 (125,811, 89.95%)	1115 (94,199, 41.83%)

Note: Numbers in table were expressed as number of MOTUs (Abundance of these MOTUs, proportion of all MOTUs)

## Discussion

Given the critical importance of metabarcoding primers in eDNA based biodiversity detection, a pair of primer with good performance is essential for environmental DNA research [27]. However, despite the increasingly wide application of eDNA metabarcoding in mollusk community surveys across diverse ecosystems, the effective identification of all mollusk species is still poorly known. In this study, seven pairs of primers were developed and tested in *in silico* PCR and PCR amplification with genomic and environmental DNA. Newly developed primers were compared with published primers to identify the most effective candidate primer for marine mollusk biodiversity surveys. Among the newly-developed primers, MollCOI253 was found to be the best candidate, demonstrating good specificity and universality for marine mollusks. This primer is expected to provide valuable technical support for mollusk biodiversity surveys, and it would be also beneficial for their conservation in the range of Chinese sea.

### Comparison of different methods for assessing primer effects

Although the *in silico* method allowed for rapid and high-volume screening of amplified species [28], the primer binding was tolerant of certain mismatches, especially in the 5′ end region [29], which could lead to discrepancies between actual amplification and *in silico* PCR results. This was further confirmed in the present study. For example, 16SrRNA showed strong specificity in the *in silico* PCR analysis, but non-specific amplification was found in genomic amplification. In contrast, Moll12S100 and Moll12S195 primers amplified a higher number of species than MollCOI253 in the *in silico* PCR analysis, while the former two amplified fewer species than the latter in the genomic amplification. Furthermore, the amplifications of eDNA showed that the performance of primers was also different from *in silico* PCR tests, with the primer that amplified the most MOTUs being Moll12S150, which was consistent with the genomic amplification results but not with the *in silico* PCR results.

Despite some differences in the assessment of specificity, universality and robustness among the three methods, all methods confirmed the presence of amplification preferences for most tested primers. For example, Moll12S100 and Moll12S195 favored gastropods and bivalves in both *in silico* PCR and genomic DNA amplification tests, whereas MollCOI253 amplifies more Polyplacophora in both *in silico* tests and environmental DNA amplification compared to the other primers. Therefore, the disagreements and agreements between these methods suggested that eDNA primer performance assays may need to be evaluated comprehensively by a combination of multiple methods.

### Comparison of designed and published primers

The primer requirements for environmental DNA metabarcoding techniques usually depend on their amplification specificity and universality [30]. In the present study, MollCOI154, MollCOI255, COI204, Sepi, and veneroida primers exhibited non-target amplifications in *in silico* PCR amplifications. Additionally, 16 S rRNA primer was further excluded from genomic DNA amplification due to cross-amplification in non-molluscan species. This suggests that these primers lacked specificity and may result in Type I errors, amplifying non-target DNA from samples that contain no eDNA of the target species [30], thus impacting the accuracy of biodiversity estimates.

On the other hand, when considering universality, Moll12S150 and NZMS amplified a much lower number of species than the other primers. Also, the primers NADH, Moll12S150, 16 S rRNA, Ceph18S and veneroida, did not perform well in terms of universality and only amplified a certain taxon. Genomic DNA amplification revealed that Moll12S195, Moll12S100 and Moll16S failed to amplify across all the tested samples. These limited coverages of amplified species had the potential to bias biodiversity estimates due to primer binding preferences [30]. After different assessments, MollCOI253 was superior to the other primers considering both specificity and species amplification coverage (Table 5).

Whether a barcode region contains an appropriate conservative sequence region directly determines whether primers based on that region can amplify all taxa. If there is no conservative region within the region, a proportion of taxa may remain unidentified following amplicon-based metabarcoding [31]. Although previous studies reported that “universal” *COI* primers that amplify barcoding regions anneal to primer-binding sites that were poorly conservative across gastropods [32], the primers designed in this study based on the *COI* region showed relatively good coverage for *in silico* amplification and genomic DNA amplification in gastropods, suggesting that the *COI* region remains a viable candidate for metabarcoding in mollusks. Although it could not be excluded that it is related to the conservativeness of the region, the relatively low number of reads amplified by MollCOI253 compared to other primers may be related to the length and size of the DNA fragments it amplifies [19]. MollCOI253 amplified a longer DNA fragment compared to the amplification products of the other primers, and therefore may yield relatively fewer reads at similar sequencing volumes. Although longer DNA fragments were easier to degradation in nature, it is clear from the results that MollCOI253 amplifies fewer but more efficient reads under the same conditions [33]. Degenerate primers containing degenerate base pairs in the primer sequences were expected to increase the species of amplification and improve primer performance [41], but this may require the selection of non-conservative regions for primer re-design in the future.

**Table 5:** Summary of all primers’ faults in application of environmental DNA

Primer name	in silico PCR	Genomic DNA PCR	Environmental DNA PCR	High-throughput sequencing
MollCOI154	Cross amplification	-	-	-
MollCOI253				
MollCOI255	Cross amplification	-	-	-
Moll12S150	Limited coverage			
Moll12S195		Partial amplification		
Moll12S100		Partial amplification		Annotation failure
Moll16S		Partial amplification	Amplification failure	-
COI204	Cross amplification	-	-	-
NADH	Limited coverage	-	-	-
NZMS	Poor coverage	-	-	-
Sepi	Cross amplification	-	-	-
16 S rRNA	Limited coverage	Cross amplification	-	-
Ceph18S	Limited coverage	-	-	-
Unionoida	Limited coverage	-		Annotation failure
Veneroida	Cross amplification	-		Annotation failure

Note: - meant analyses were not conducted in this study

### Annotation effects of MollCOI253 on eDNA samples

The highest percentage of annotations was from the amplification of MollCOI253, which accounted for only 11.78% of the reads. Compared with 16SrRNA in previous studies, the ratio of annotation of mollusks by MollCOI253 was lower than 26.69% of 16SrRNA [15]. This small proportion of annotated MOTUs could be explained by the incompleteness of reference databases [34]. Currently, the reference sequences used in most mollusk metabarcoding analysis were from the NCBI database [35], and insufficient sequence data could result in low efficiency of mollusk classification [36]. Therefore, to use eDNA technology for more accurate and effective studies of mollusk biodiversity, further efforts are needed to establish a more complete reference sequence database of mollusks.

In this study, some common East China Sea mollusk species were undetected in the eDNA samples (e.g., *Rapana bezoar*, *Coelomactra antiquate*, *Octopus vulgaris*), and the reason for this may be related to the fact that eDNA of these mollusks were not collected, failed to be amplified, or were not annotated [5]. Therefore, further improvements in eDNA extraction [37], amplifications [38], and annotated reference database [36] should be made in the future. For instance, adding proteinase K could significantly improve DNA extraction [39], and the use of multiple primers for amplification could also improve the accuracy and detection rate of eDNA technology [38] .

Among the annotated results, two exotic species, *Powelliphanta patrickensis* and *Bursa scrobilator*, were found, both of which have never been reported offshore China [3]. Given their extremely low abundance (0.003-0.037%), it was possible that they were artificially transported to the sampling area (e.g., ballast water), or that they are recently introduced alien species. Also, it could not be excluded that it was the result of errors in the sequencing and assembly process. Although further investigation of MOTUs was needed for these species, these very low abundance or occasional MOTUs were often excluded from community diversity analyses [5]. Therefore, these unreported low abundance species did not have a significant impact on the results of community diversity analyses, especially beta diversity. However, if these species needed to be detected, it would be recommended that more specific primers should be designed for better detection.

## Conclusions

Seven primer pairs were designed based on the *COI*, *12 S* and *16 S* genes in this study, of which MollCOI253, as the best candidate, outperformed other newly designed and published primers for molluscan eDNA studies in terms of versatility, specificity and robustness. The results of this study not only provided technical support for molluscan eDNA investigations, but also provide a reference for the selection of multiple molecular markers for future molluscan eDNA studies. The results of this study will be helpful for more effective investigations of molluscan biodiversity in the future.

### Electronic supplementary material

Below is the link to the electronic supplementary material.

Supplementary Material 1

## Data Availability

Raw sequencing data from the MollCOI253 (SRR27338740-41, SRR27338732-33), Moll12S150 (SRR27338728-31), Moll12S195 (SRR27338726-27), Moll12S100 (SRR27338738-39), unionoida (SRR27338736-37) and veneroida (SRR27338734-35) sequencing are available in the NCBI SRA database under bioproject (PRJNA1049577).
