# Supplementary material for: Development of environmental DNA metabarcoding primers for marine mollusks and comparison with published primers
Source: BMC Ecol Evol. 2024 May 31;24:73. doi: 10.1186/s12862-024-02265-8 (PMC11140855; doi:10.1186/s12862-024-02265-8)
Supplement: Supplementary file 1 — Supplementary Material 1 [file 12862_2024_2265_MOESM1_ESM.docx]

**Development of environmental DNA metabarcoding primers for marine mollusks and comparison with published primers**

Xiaojing Shi^a^, Yihui Jiang^a^, Ling Cao^b^, Cong Zeng^a,*^

a School of Oceanography, Shanghai Jiao Tong University, Shanghai 200030, China

b State Key Laboratory of Marine Environmental Science, College of Ocean and Earth Sciences, Xiamen University, Xiamen 361104, China

* Corresponding author: Cong Zeng, congzeng@sjtu.edu.cn

Table S1 Numbers of amplified species from *in silico* PCR results with newly developed and published primers

| Primer name | Aplacophora | Cephalopoda | Polyplacophora | Bivalvia | Gastropoda | Non-mollusk |
| --- | --- | --- | --- | --- | --- | --- |
| References | 0 | 27 (13%) | 0 | 125 (59%) | 61 (29%) | 0 |
| MollCOI154 | 1 (1%) | 2 (1%) | 1 (1%) | 51 (26%) | 118 (60%) | 25 (13%) |
| MollCOI253 | 7 (7%) | 4 (4%) | 39 (37%) | 20 (19%) | 36 (34%) | 0 |
| MollCOI255 | 0 | 2 (1%) | 2 (1%) | 39 (11%) | 241 (70%) | 61 (18%) |
| MOll12S150 | 0 | 0 | 0 | 23 (92%) | 2 (8%) | 0 |
| MOll12S195 | 0 | 11 (8%) | 2 (1%) | 123 (89%) | 2 (1%) | 0 |
| MOll12S100 | 0 | 1 (0%) | 0 | 10 (3%) | 300 (96%) | 0 |
| Moll16S | 0 | 0 | 0 | 139 (99%) | 2(1%) | 0 |
| Ceph18S | 0 | 152 (100%) | 0 | 0 | 0 | 0 |
| 16SrRNA | 0 | 0 | 0 | 172 (100%) | 0 | 0 |
| unionida | 0 | 0 | 0 | 102 (100%) | 0 | 0 |
| veneroida | 0 | 0 | 0 | 106 (83%) | 18 (14%) | 3 (2%) |
| Sepi | 0 | 35 (56%) | 0 | 1 (2%) | 25 (40%) | 1 (2%) |
| COI204 | 0 | 26 (21%) | 1 (1%) | 2 (2%) | 62 (51%) | 31 (25%) |
| NADH | 0 | 0 | 0 | 57 (100%) | 0 | 0 |
| NZMS | 0 | 0 | 0 | 1 (25%) | 3 (75%) | 0 |

Table S2 Mollusk species for testing primers using genomic DNA

| Class | Family | Species | 16SrRNA | MollCOI253 | Moll12S150 | Moll12S195 | Moll12S100 | Moll16S |
| --- | --- | --- | --- | --- | --- | --- | --- | --- |
| Gastropoda | Haliotidae | Haliotis discus | √ | √ | √ | √ | √ | √ |
|  | Littorinidae | Nodilittorina pyramidalis | √ | √ | √ | √ | √ | × |
|  | Babyloniidae | Babylonia lutosa | √ | √ | √ | × | √ | × |
|  | Buccinidae | Neptunea cumingii | √ | √ | √ | × | √ | × |
|  | Muricidae | Rapana bezoar | √ | √ | √ | √ | √ | √ |
|  |  | Rapana rapiformis | √ | √ | √ | √ | √ | √ |
| Bivalvia | Mactridae | *Mactra quadrangularis* | √ | √ | √ | × | × | × |
|  |  | *Mactra veneriformis* | √ | √ | √ | × | × | × |
|  |  | Mactra antiquata | √ | √ | √ | × | × | × |
|  |  | *Mactra chinenesis* | √ | √ | √ | × | × | × |
|  | Arcidae | *Tegillarca granosa* | √ | √ | √ | √ | √ | √ |
|  |  | Scapharca subcrenata | √ | √ | √ | √ | × | × |
|  | Veneridae | *Paphia undulata* | √ | √ | √ | √ | √ | × |
|  |  | Meretrix meretrix | √ | √ | √ | √ | √ | √ |
|  | Ostreidae | Grassostrea gigas | √ | √ | √ | √ | × | × |
|  | Pectinidae | Azumapecten farreri | √ | √ | √ | √ | × | √ |
|  | Mytilidae | Mytilus edulis | √ | √ | √ | √ | × | √ |
|  |  | Perna viridis | √ | √ | √ | √ | × | √ |
|  |  | Musculus senhousei | √ | √ | √ | √ | × | √ |
|  |  | Trichomya hirsuta | √ | √ | √ | √ | × | √ |
|  | Pharidae | Sinonovacula constricta | √ | √ | √ | √ | × | × |
|  | Solenidae | Solen strictus | √ | √ | √ | √ | × | × |
| Cephalopoda | Octopodidae | Octopus vulgaris | √ | √ | √ | × | √ | × |
|  | Loliginidae | Loligo chinensis | √ | √ | √ | × | √ | × |
| Decapoda | Penaeidae | *Penaeus monodon* | √ | × | × | × | × | × |
|  | Ocypodidae | *Ocypoda ceratophthalma* | √ | × | × | × | × | × |

Table S3 Numbers of amplified species from *in silico* PCR results with newly developed and published primers

| Primer name | Name | Superclass | Species | Abundance | Proportion/% |
| --- | --- | --- | --- | --- | --- |
| MollCOI253 | MOTU10, MOTU14, MOTU180, MOTU305, MOTU426, MOTU567 | Gastropoda | *Sulcospira paludiformis* | 4584 | 3.926 |
|  | MOTU7 | Polyplacophora | *Mopalia spectabilis* | 4138 | 3.544 |
|  | MOTU22 | Gastropoda | *Choanomphalus hyaliniiformis* | 2827 | 2.421 |
|  | MOTU90, MOTU356 | Gastropoda | *Gyraulus* sp | 745 | 0.638 |
|  | MOTU46 | Gastropoda | *Eatoniella atropurpurea* | 581 | 0.498 |
|  | MOTU58 | Bivalvia | *Corbicula fluminea* | 371 | 0.318 |
|  | MOTU87 | Bivalvia | *Laternula* sp | 240 | 0.206 |
|  | MOTU252 | Gastropoda | *Valvata utahensis* | 141 | 0.121 |
|  | MOTU156 | Gastropoda | *Frolikhiancylus frolikhae* | 70 | 0.060 |
|  | MOTU205 | Gastropoda | *Powelliphanta patrickensis* | 43 | 0.037 |
|  | MOTU362 | Gastropoda | *Biomphalaria obstructa* | 6 | 0.005 |
|  | MOTU410 | Gastropoda | *Bursa scrobilator* | 3 | 0.003 |
| Moll12S150 | MOTU136, MOTU2530, MOTU3478, MOTU4908, MOTU4953, MOTU5190, MOTU5881, MOTU6160, MOTU6477, MOTU7317, MOTU7326, MOTU8410, MOTU9713, MOTU10073 | Bivalvia | *Crassostrea ariakensis* | 400 | 0.121 |
|  | MOTU3764 | Gastropoda | *Radix acuminata* | 11 | 0.003 |
|  | MOTU3187 | Cephalopoda | *Doryteuthis opalescens* | 7 | 0.002 |
| Moll12S195 | MOTU445 | Bivalvia | Lucinidae | 9 | 0.002 |
